# Mechanistic Insights into the Antioxidant Potential of Sugarcane Vinegar Polyphenols: A Combined Approach of DPPH-UPLC-MS, Network Pharmacology and Molecular Docking

**DOI:** 10.3390/foods13213379

**Published:** 2024-10-24

**Authors:** Feifei Wu, Bo Lin, Jing Chen, Fengjin Zheng, Yuxia Yang, Usman Rasheed, Ganlin Chen

**Affiliations:** 1 Guangxi South Subtropical Agricultural Research Institute, Guangxi Academy of Agricultural Sciences, Longzhou 532400, China; wufeifei@gxaas.net (F.W.); jchen1030@gxaas.net (J.C.); 2 Institute of Agro-Products Processing Science and Technology, Guangxi Academy of Agricultural Sciences, Nanning 530007, China; linbo@gxaas.net (B.L.); zhengfengjin@gxaas.net (F.Z.); yangyuxia@gxaas.net (Y.Y.); 3 Guangxi Subtropical Crops Research Institute, Guangxi Academy of Agricultural Sciences, Nanning 530001, China; rasheus@outlook.com; 4 Key Laboratory of Quality and Safety Control for Subtropical Fruit and Vegetable, Ministry of Agriculture and Rural Affairs, Nanning 530001, China; 5 Guangxi Key Laboratory of Quality and Safety Control for Subtropical Fruits, Nanning 530001, China

**Keywords:** sugarcane vinegar polyphenols, antioxidants, oxidative stress, DPPH-UPLC-MS, network pharmacology, molecular docking

## Abstract

This study investigated the antioxidant potential of sugarcane vinegar, an emerging functional food, by analyzing its polyphenols and underlying molecular mechanisms that intervene in oxidative stress. Using a 1,1-diphenyl-2-trinitrophenylhydrazine (DPPH) assay combined with UPLC-MS analysis, six key polyphenols were identified: chlorogenic acid, caffeic acid, ferulic acid, luteolin, protocatechuic acid, and syringic acid. These compounds showed a positive correlation with antioxidant capacity. In a simulated sugarcane vinegar environment, these polyphenols exhibited synergistic antioxidant effects, while in methanol, antagonistic interactions were predominant. Network pharmacology revealed five key polyphenols targeting 10 critical proteins involved in oxidative stress, including the PI3K-Akt and IL-17 signaling pathways. Molecular docking confirmed strong binding affinities between these polyphenols and core targets like PTGS2, STAT3, and GSK3B. This study establishes a reference for the antioxidant mechanisms of sugarcane vinegar and highlights its potential for developing functional products.

## 1. Introduction

Fruit vinegars are produced from fruits or fruit juices through a two-stage process of alcoholic and acetic acid fermentation and are popular among consumers due to their associated health benefits [1,2]. It is well-documented that fruit vinegars possess anti-inflammatory, anti-aging, lipid-lowering, and cardiovascular disease prevention effects, largely attributed to their antioxidants and ability to modulate oxidative stress [3,4,5]. The phenolic composition and concentration are crucial factors influencing the antioxidant activity of fruit vinegar, with raw fruit materials serving as the primary source of these phenolic compounds. Consequently, the antioxidant capacity and mechanism of action vary across different fruit vinegars [3,4]. Sugarcane vinegar is a relatively new type of fruit vinegar, produced from sugarcane juice through a two-step process of alcoholic and acetic acid fermentation [1]. In a previous study by our group [6], it was discovered that sugarcane vinegar beverages, diluted and formulated from sugarcane vinegar, contain various phenolic compounds, including chlorogenic acid, caffeic acid, ferulic acid, and luteolin. Compared to six commercially available apple vinegar drinks, sugarcane vinegar drinks exhibited significantly higher levels of phenolic acids, with the ferulic acid content being 81.87–83.66% higher and caffeic acid content 78.28–94.83% higher. Furthermore, it was found that sugarcane vinegar effectively scavenged 1,1-diphenyl-2-trinitrophenylhydrazine (DPPH) free radicals and alleviated oxidative stress symptoms in hyperlipidemic mice [1]. However, despite the presence of various phenolic compounds in sugarcane vinegar, the specific contributions of individual phenolics to its overall antioxidant activity remain unclear.

DPPH is a stable free radical that reacts with the active hydrogen atoms of antioxidant substances, leading to a reduction in the concentration of antioxidant active components. Conversely, substances without antioxidant activity do not react with DPPH, and their contents remain unchanged [7]. Therefore, the ability of a compound to scavenge DPPH can be accessed by measuring the changes in its concentration before and after reaction with DPPH [7,8]. Given that high-performance liquid chromatography (HPLC) and liquid chromatography–mass spectrometry (LC-MS) are commonly used to analyze phytochemical components, combining the DPPH method with HPLC and LC-MS can rapidly identify antioxidant active components in a sample. The DPPH method, coupled with HPLC and LC-MS for the identification of the antioxidant active components in samples, is envisioned as a low-cost, simple-to-operate, and highly sensitive detection technique [9,10]. Our previous research developed a UPLC-MS method [11] for analyzing sugarcane vinegar polyphenols. Based on that, the DPPH method combined with UPLC-MS (DPPH-UPLC-MS) could effectively screen the primary antioxidant active components of sugarcane vinegar polyphenols.

Network pharmacology is an emerging discipline grounded in the principles of systems biology, which challenges the traditional “one drug, one disease” paradigm [12,13]. Constructing a complex network of herbal compounds and their targets enables a systematic description of the intricate interactions among biological systems, drugs, and diseases [13]. The majority of network pharmacology studies on herbal medicines rely on herbal compounds found in online public databases [14,15]. However, these studies cannot ensure that the compounds used for network pharmacology analyses are actually the active substances present in the herbal preparations [15]. Meanwhile, docking is a technique that involves spatially fitting a small molecule to a large molecule receptor and scoring the complementarity at the binding site, thereby helping to predict the compound’s binding affinity to its target [15]. In applying this, by screening the main active components of a polyphenol mixture for antioxidant activity using the DPPH-UPLC-MS method, in combination with network pharmacology analysis and molecular docking, we can ascertain more reliable pharmacological targets for these polyphenols.

The objective of this study was to investigate the antioxidant potential of sugarcane vinegar by analyzing its polyphenolic composition and the molecular mechanisms involved in combating oxidative stress. By identifying key polyphenols and their interactions with critical oxidative- stress-related proteins, the study aimed to provide insights into the health benefits of sugarcane vinegar and its potential applications in developing functional food products. The potential targets, biological processes (BPs), and signaling pathways of sugarcane vinegar polyphenols in combating oxidative stress were identified using network pharmacology tools. A “compound–core target–signaling pathway” network was constructed to illustrate the integrated interactions and mechanisms of these polyphenols. At the same time, molecular docking was employed to assess the binding stability of the key active ingredients with core targets related to oxidative stress. To our knowledge, this is the first comprehensive study to elucidate the potential molecular mechanisms of the antioxidants in sugarcane vinegar polyphenols using a combination of DPPH-UPLC-MS, network pharmacology, and molecular docking. This study provides a scientific foundation and theoretical guidance for the quality evaluation of sugarcane vinegar, the development of functional products, and the regulation of functional components within these products.

## 2. Materials and Methods

### 2.1. Chemicals and Reagents

Analytical-grade standards of rutin (RT) and gallic acid (GA), Trolox standards, and HPLC-grade standards of apigenin (AG), caffeic acid (CA), cinnamic acid (CMA), p-coumaric acid (pCA), ferulic acid (FA), luteolin (L), protocatechuic acid (PA), salicylic acid (SCA), syringic acid (SA), chlorogenic acid (CGA), and vanillic acid (VA) were purchased from Shanghai YuanYe Biotechnology Co., Ltd. (Shanghai, China); HPLC-MS-grade acetonitrile and formic acid were purchased from Fisher Scientific; analytically pure 1,1-diphenyl-2-trinitrophenylhydrazine (DPPH), 2,2-azidobis-(3-ethylbenzodihydrothiazoline-6-sulfonic acid) diammonium salt (ABTS), sodium acetate, and Folin–Ciocalteu reagent were acquired from Solarbio (Beijing, China); methanol, ethyl acetate, glacial acetic acid, and other chemicals (analytical grade) were purchased from Chengdu Colony Chemical Co.; brewer’s yeast was purchased from Angie’s Yeast Co., Ltd., Yichang, China; and *Lactobacillus* (LB) was purchased from Shanxi Dingfeng Brewing Technology Co., Ltd., Xianyang, China. Sodium acetate buffer (pH 3.3) was prepared by dissolving 5.1 g of sodium acetate and 20 mL of glacial acetic acid, diluting it to 250 mL with purified water, and then adjusting the pH to 3.3 with glacial acetic acid.

### 2.2. Sample Preparation

Sugarcane vinegar was prepared following our previous method [11]. Sugarcane juice was adjusted to 20° Brix with sugar, inoculated with 10 g/L of fruit wine yeast (Angie’s Yeast Co., Ltd., Yichang, China), and fermented at 27 °C for 18 days. The fermentation broth was then diluted 2-fold with sterile water, supplemented with 5 g/L of LB acetate bacteria, and fermented at 25 °C for 20 days. Finally, the broth was filtered and sterilized at 80 °C for 30 min to produce sugarcane vinegar.

Sugarcane vinegar extract was prepared by concentrating sugarcane vinegar (200 mL of raw vinegar in five portions) to a viscous consistency with a rotary evaporator (55 °C). Then, it was immersed in 50 mL of pure water, methanol, ethanol, n-butanol, or ethyl acetate for 24 h, followed by centrifugation at 2599× *g* for 10 min to collect the extracts. This extraction procedure was performed twice, and extracts were pooled and dried in a rotary evaporator at 55 °C. Finally, the extracts were dissolved in 25 mL of 70% ethanol and stored at 4 °C.

### 2.3. Determination of Total Phenolic Content and Total Flavonoids

The total phenolic content (TPC) analysis was carried out whereby the TPC was determined by the Folin–Ciocalteu method adopted from Özdemir et al. [16] and Hu et al. [17]. Briefly, 150 μL of the diluted sample was mixed with 750 μL of 10% Folin–Ciocalteu reagent (stood for 5 min) and 600 μL of 7.5% (*w*/*v*) Na_2_CO_3_. The mixture was placed in the dark for 1.5 h. The absorbance was read at 760 nm, and TPC was calculated as gallic acid equivalents (mg GAE/mL).

The total flavonoid content (TFC) was determined as described by Bai et al. [18]. Briefly, an appropriately diluted sample was combined with 0.7 mL of 5% NaNO_2_ and kept at room temperature for 6 min, followed by the addition of 0.7 mL of 10% Al (NO_3_)_3_. The mixture was left to stand for 6 min before adding 2 mL of 4% NaOH and adjusting to 10 mL with ethanol (60%). After incubation at room temperature for 10 min, the absorbance value was measured at 510 nm. The flavonoid content was expressed as mg of rutin equivalent per mL of sugarcane vinegar (mg RE/mL).

### 2.4. Screening Active Compounds by DPPH-UPLC-MS

The aqueous extract of sugarcane vinegar was prepared according to the method mentioned in the previous section. After evaporating the solvent, the extract was re-constituted in 5 mL of pure methanol. To prepare the samples for analysis, 1 mL of the methanol solution of sugarcane vinegar extract was mixed with 1 mL of the methanolic solution of DPPH (15 mg/mL). The mixture was allowed to react in the dark for 30 min, centrifuged for 10 min at 10,000 rpm, filtered through 0.22 μm micron syringe filters (PTFE, Biosharp, Beijing, China), and stored at a low temperature until analysis.

The polyphenolic compounds of sugarcane vinegar in the samples were analyzed by a Waters EVEO TQ-S ultrahigh-pressure liquid chromatograph equipped with an electrospray ionization source (Waters Technology Shanghai Co., Ltd., Shanghai, China). The control sample was prepared by mixing 1 mL of methanol with 1 mL of sugarcane vinegar extract. The chromatographic conditions for the detection of phenolic compounds were as follows: chromatographic column: ACQUITY UPLC@ HSS T3 (2.1 mm×100 mm, 1.7 μm, Waters USA); mobile phase A: 0.1% formic acid in ultrapure water, mobile phase B: 100% acetonitrile solution, flow rate of 0.25 mL/min using a gradient elution program; 0~0.80 min, 90% A; 0.8~4.0 min, 90%~80% A; 4.0~6.5 min, 80% A; 6.5~7.0 min, 80%~55% A; 7.0~13.0 min, 55% A; 13.0~13.5 min, 55%~90% A; 13.5~14.0 min, 90% A, while the column temperature was kept at 35 °C and 2 μL of the sample was injected for analysis. The mass spectrometric conditions were adapted as follows: electrospray ionization source (ES+, ES−), scanning mode of multiple reaction monitoring (MRM), capillary voltage: 3.0 KV, ionization source temperature: 350 °C, desolvation gas (N_2_), flow rate of desolvation gas: 700 L/h, flow rate of cone gas: 150 L/h, flow rate of collision gas (Ar): 0.10 mL/min.

The consumption rate (%) of bioactive phenolic compounds was calculated according to the following equation:

*Consumption rate* (%) = (*M_After_* − *M_Before_*)/*M_Before_*, (1)

*M_Before_*: the contents of polyphenolic compounds in mixed samples of sugarcane vinegar extract and DPPH solution.

*M_After_*: the contents of polyphenolic compounds in control samples.

### 2.5. Antioxidant Capacity of Polyphenol Compounds of Sugarcane Vinegar

#### 2.5.1. Determination of DPPH Radical Scavenging Capacity

A DPPH free radical scavenging capacity assay was implemented according to a previously published procedure [19] with some modifications. Briefly, 100 µL of DPPH methanolic solution (100 µg/mL) was mixed with 100 µL of varying concentrations of samples in a 96-well plate. The mixed reaction solution was incubated for 6 min under dark conditions, with no exposure to light, and subsequently measured at a wavelength of 734 nm using a BioTek Epoch microplate reader (BioTek, Shoreline, WA, USA). A mixture of 100 µL of DPPH solution and 100 µL of solution was used as a control, and the radical scavenging ability was calculated as Trolox equivalent TEAC (mg TE/100 mL).

To determine the antioxidant capacity of individual polyphenol compounds in sugarcane vinegar, solutions of varying concentrations of each compound standard were prepared, ensuring that the absorbance values fell within the range of 0.2–0.8. These solutions were then assayed following the previously described method. Finally, the IC50 values (µg/mL) of each compound were calculated to quantify their antioxidant activity.

#### 2.5.2. Determination of ABTS Radical Scavenging Capacity

ABTS free radical scavenging capacity was determined according to Boasiako et al. [20]. Briefly, 150 µL of the working solution was mixed with 50 µL of samples of different concentrations in a 96-well plate. The mixed reaction solution was reacted for 6 min in the dark followed by detection at a wavelength of 734 nm using a BioTek epoch (BioTek, USA). The results were presented as Trolox equivalent TEAC (mg TE/100 mL).

#### 2.5.3. Antioxidant Capacity of the Binary Interactions Among Phenolic Compounds

The Chou–Talalay method, also known as the combination index method and median potency method, is used to evaluate the interaction of two drugs through the combination index (CI) [21]. Referring to the method of Chou [21], the respective solutions of IC50 (µg/mL) of each polyphenol compound standard (CGA, CA, FA, L, SA, and PA) of the sugarcane vinegar were prepared at concentrations of 0.1 × IC50, 0.25 × IC50, 0.5 × IC50, 0.75 × IC50, 1.0 × IC50, 1.25 × IC50, 1.5 × IC50, 1.75 × IC50, and 1.75 × IC50, respectively. Solutions of two compounds were mixed at the same level of concentration in a 1:1 volume ratio, and their DPPH scavenging ability was determined according to Section 2.5.1. The combination index was calculated according to the following equation:

*CI* = D_1_/D_χ1_ + D_2_/D_χ2_, (2)

where D_1_ and D_2_ represent the concentrations required for each of the compounds to interact to produce the X effect, while D_χ1_ and D_χ2_ denote the concentration required for each of the compounds to produce the X effect. The combination index was represented as follows: where *CI* < 1 indicates a synergistic effect (the smaller the value, the stronger the synergy); *CI* = 1 indicates an additive effect; and *CI* > 1 indicates an antagonistic effect (the larger the value, the stronger the antagonism).

Comprehensive measurements of compound interactions were calculated as the weighted average combination index. Different ranges of values of *CI*_wt_ express varying degrees of antioxidant interactions. A lower *CI*_wt_ value reflects a stronger synergistic antioxidant interaction synergy, as detailed in Table 1.

*CI*_wt_ = (*CI*_50_ + 2*CI*_75_ + 3*CI*_90_ + 4*CI*_95_)/10, (3)

where *CI*_50_ represents the *CI* value at a 50% action concentration and similarly for *CI*_75_, *CI*_90_, and *CI*_95_.

### 2.6. Network Pharmacology

#### 2.6.1. Screening Candidate Compounds and Potential Targets

The SDF files of the 2D structures of the sugarcane vinegar polyphenol compounds were retrieved from the PubChem database (https://pubchem.ncbi.nlm.nih.gov/ (accessed on 6 April 2024)) and then submitted to the Swiss Target Prediction tool (http://swisstargetprediction.ch/ (accessed on 6 April 2024)) and the TCMSP (https://old.tcmsp-e.com/tcmsp.php (accessed on 6 April 2024)) to predict the targets of these compounds. The target information obtained was normalized using the UniProt database (https://www.uniprot.org/ (accessed on 7 April 2024)), while the species was specified as “Homo sapiens”.

Using “Oxidative Stress” as the keyword, searches were performed in the GeneCards database (https://www.genecards.org/ (accessed on 10 April 2024)), OMIM database (https://www.omim.org/ (accessed on 10 April 2024)), PharmGKB database (https://www.pharmgkb.org/ (accessed on 10 April 2024)), TTD database (https://db.idrblab.net/ttd/ (accessed on 10 April 2024)), and DrugBank database (https://go.drugbank.com/ (accessed on 10 April 2024)) for targets related to oxidative stress. The targets collected from these five databases were organized and compiled.

The Venn Diagram package in R was utilized to identify the intersection between sugarcane vinegar polyphenols and oxidative-stress-related targets. The intersecting targets, along with their corresponding sugarcane vinegar polyphenols, were used to construct a sugarcane vinegar polyphenols–oxidative stress target network diagram using Cytoscape 3.7.0 software. The importance of each node within the network was assessed based on its degree value, with a higher degree value indicating a more critical role in the network [22].

#### 2.6.2. Pathway Enrichment Analysis

Gene Ontology (GO) is an internationally recognized system for classifying gene functions. The intersecting gene targets identified in Section 2.6.1 were subjected to GO functional enrichment analysis using the Bioconductor org.Hs.eg.db package in R. The top 10 entries, in terms of biological process (BP), cellular component (CC), and molecular function (MF), that were most closely associated with oxidative stress (*p* < 0.05) were selected for visualization. KEGG (Kyoto Encyclopedia of Genes and Genomes) analysis is a powerful tool for exploring the biological pathways and functions of target genes, offering insights into molecular interactions and response networks [15]. KEGG pathway analysis was conducted using the DAVID online platform (https://david.ncifcrf.gov/ (accessed on 28 April 2024)). The top 10 pathways (*p* < 0.05) associated with oxidative stress were selected for visual representation.

#### 2.6.3. Protein–Protein Interaction Network Construction and Screening of Core Targets

The protein–protein interaction network (PPI network) was constructed using the STRING database (https://cn.string-db.org/ (accessed on 30 April 2024)) for the 30 GO functional terms and 10 KEGG signaling pathway-related targets identified in Section 2.6.2, while the species was set to “Homo sapiens”. The remaining parameters were employed with default values. Target node data were retrieved, and Cytoscape 3.7.0 was used to create the network diagram. Targets with values greater than the median for the six topological parameters (Betweenness, Closeness, Degree, Eigenvector, LAC, and Network) were screened. This screening process was conducted twice to ensure the robustness of the selected targets.

#### 2.6.4. Molecular Docking Analysis

Molecular docking was performed according to a published procedure [15]. Briefly, the 2D structures of sugarcane vinegar polyphenolic compounds (CGA, CA, FA, L, PA) were retrieved from the PubChem database (https://pubchem.ncbi.nlm.nih.gov/ (accessed on 8 May 2024)). Their 3D structures were generated and energy-minimized using ChemDraw 19.0 software. Protein conformations of core targets (PTGS2, STAT3, HSP90AA1, MMP9, RELA, GSK3B, AR, MET, CDK2, and NFE2L2) were obtained from the Protein Data Bank (PDB, https://www.rcsb.org/ (accessed on 8 May 2024)). Molecular docking between the core targets and the polyphenolic compounds was performed using Autodock tools to find the lowest binding energy for each molecular group. The docking results were visualized and analyzed with Pymol 2.3.2 software.

### 2.7. Data Analysis

Data were presented in triplicate, and the results were expressed as the mean ± standard deviation. Statistical analyses were conducted using one-way analysis of variance (ANOVA), followed by Tukey’s test for multiple comparisons (*p* < 0.05). Correlation analyses between variables were also performed using IBM SPSS Statistics v23.

## 3. Results and Discussion

### 3.1. Correlation of TEAC, TPC, and TFC Values

#### 3.1.1. Effects of Extraction Solvents on TPC and TFC of Sugarcane Vinegar Extracts

**Figure 1:**
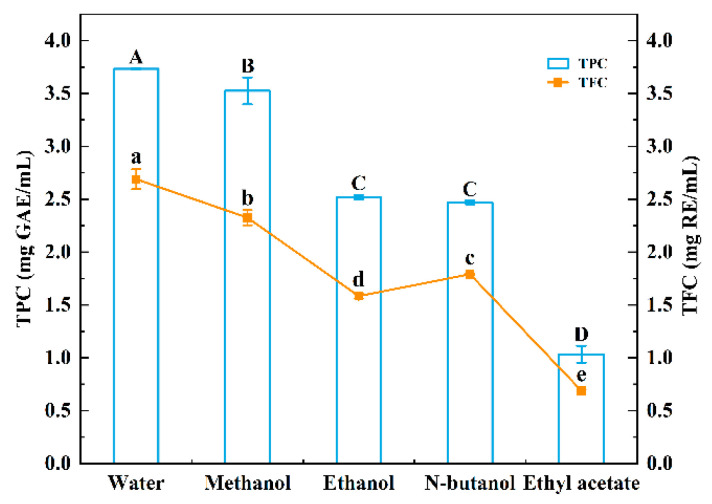
Effect of extraction solvents on total phenolic content (TPC) and total flavonoid content (TFC) of sugarcane vinegar extracts. Different uppercase and lowercase letters indicate significant differences of TPC and TFC at *p*-value < 0.05.

The choice of extraction solvent directly influences the efficiency, type, and yield of polyphenols extracted from food products. Selecting an appropriate solvent is crucial for maximizing the extraction of polyphenols [23]. As shown in Figure 1, the total flavonoid content (TFC) of sugarcane vinegar extracts using different solvents was lower than the total phenolic content (TPC). This suggests that the polyphenols in these sugarcane vinegar extracts include not only flavonoids but also other types of phenolics. The polarity ranking of the four solvents used in this study was as follows: water > methanol > ethanol > N-butanol > ethyl acetate. The total phenolic content in the sugarcane vinegar extracts showed a decreasing trend with decreasing solvent polarity. This observation aligns with Arzola-Rodríguez et al., that most natural antioxidants derived from plants, such as phenolic acids and polyphenols, exhibit hydrophilic properties and tend to have lower solubility in less polar or hydrophobic environments [24]. In addition, most natural antioxidants derived from plants, particularly phenolic acids, typically possess hydrophilic properties. Therefore, enhancing the solubility of phenolic acids is essential for improving their bioavailability and effectiveness [25]. The water extract exhibited the highest TPC (3.73 ± 0.005 mg GAE/mL) and TFC (2.69 ± 0.093 mg RE/mL), while the ethyl acetate extract had the lowest TPC (1.033 ± 0.081 mg GAE/mL) and TFC (0.69 ± 0.006 mg RE/mL). Consequently, water was chosen as the optimal solvent for extracting polyphenols from sugarcane vinegar for subsequent studies.

#### 3.1.2. Effect of Extraction Solvents on Antioxidant Capacity of Sugarcane Vinegar Extracts

**Figure 2:**
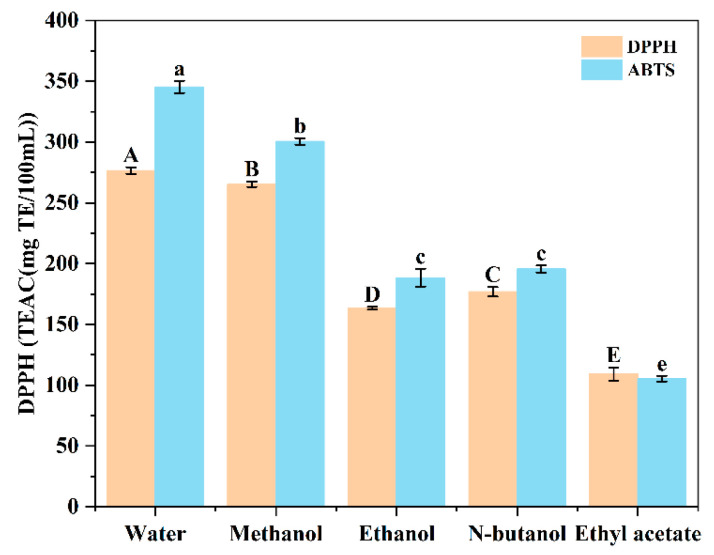
Effect of extraction solvents on antioxidant activities of sugarcane vinegar extracts. Different uppercase or lowercase letters indicate significant differences at *p*-value < 0.05.

2,2-azidobis-(3-ethylbenzodihydrothiazoline-6-sulfonic acid) diammonium salt (ABTS) and 1,1-diphenyl-2-trinitrophenylhydrazine (DPPH) assays are widely used for their quick and easy assessment of the antioxidant capacity of phenolic compounds [23,26]. Figure 2 illustrates the DPPH and ABTS scavenging capacity of different sugarcane vinegar extracts, which showed consistent trends. The reason may be that the mechanism of scavenging DPPH is similar to that of scavenging ABTS, and these two free radicals discolor themselves after reduction by accepting hydrogen atoms in antioxidants [23]. The aqueous sugarcane vinegar extract demonstrated the highest scavenging capacity, with Trolox equivalent antioxidant capacity (TEAC) values of 276.58 ± 2.64 mg TE/100 mL for DPPH and 345.39 ± 5.08 mg TE/100 mL for ABTS. The trends in TEAC values of different extracts were similar to those of their TPC and TFC. This suggests that polyphenols may be the major antioxidant components in sugarcane vinegar extracts, which requires further analysis of them.

#### 3.1.3. Correlation Analysis

Correlation analysis of TPC, TFC, and TEAC in sugarcane vinegar extracts using different solvents was conducted to further investigate the relationship between the antioxidant activity of sugarcane vinegar and its polyphenol content. As can be seen in Table 2, the correlation coefficients (R^2^) of TEAC values for TPC ascertained by DPPH and ABTS assays were 0.966 and 0.968, respectively. Similarly, the correlation coefficients of TFC produced by DPPH and ABTS assays were 0.970 and 0.978, respectively. Notably, the correlation coefficients consistently remained above 0.95 (*p*-value < 0.01), indicating a strong positive correlation. In a previous study, Lui et al. [3] reported that the antioxidant activity of fruit vinegar was positively correlated with TPC and TFC (*p* < 0.01), similar to the results of the present study. Both TPC and TFC were positively correlated with the antioxidant activity of sugarcane vinegar extracts. Given that flavonoids belong to the polyphenol class, this suggests that phenolics are the main active components of sugarcane vinegar responsible for scavenging free radicals.

### 3.2. Screening Active Compounds by DPPH-UPLC-MS

The UPLC-MS technique was employed in our previous research to detect polyphenols in sugarcane vinegar. The results revealed that 10 polyphenolic compounds exhibited high contents and good reproducibility [6,11]. This suggests that these 10 compounds may be the main phenolic compounds in sugarcane vinegar. To further investigate the contribution of polyphenolic compounds to the antioxidant activity of sugarcane vinegar extract, the aqueous extract was analyzed for previously identified major active polyphenol compounds using DPPH scavenging combined with UPLC-MS (DPPH-UPLC-MS). The results indicated that the reaction of sugarcane vinegar extract with DPPH led to variations in the concentration of phenolic compounds (Table 3). The concentrations of AG, CCA, pCA, and VA did not exhibit significant differences before and after the reaction, with depletion rates of 8%, −7.69%, −3.61%, and 2.75%, respectively. This indicates that these components are not the primary active DPPH-scavenging species in sugarcane vinegar. Similarly, a previous report on the antioxidant activity of cinnamic acid analogs showed that cinnamic acid had no DPPH scavenging activity [27]. Cinnamic acid (CMA) is a plant monophenol that can be converted to compounds such as p-coumaric acid and cinnamate esters by reactions such as hydroxylation, esterification, and methylation. In addition, cinnamate esters can be hydrolyzed to produce cinnamic acid [28,29]. P-coumaric acid is a common precursor of phenylpropanoids, lignans, flavonoids, and astragalus. P-coumaric acid can be produced through the hydrolysis of coumarin [27,30]. The negative scavenging rates of these compounds may be attributed to the increase in their content during the DPPH scavenging process, likely due to degradation and redox reactions of related polyphenols [31,32]. The concentrations of CA, FA, L, PA, SA, and CGA decreased significantly after the reaction, with depletion rates ranging from 18.37% to 100%. This indicates that these six compounds are the primary active polyphenols responsible for DPPH scavenging by sugarcane vinegar extract.

### 3.3. Antioxidant Capacity of Polyphenol Compounds of Sugarcane Vinegar

#### 3.3.1. Effect of Solvent on Antioxidant Activity of Polyphenols from Sugarcane Vinegar

The antioxidant activity of phenolics is affected by reaction conditions such as pH and solvent type in addition to their structural characteristics [33]. Sugarcane vinegar exhibits an acidic pH of 3.3. To simulate the antioxidant activity of polyphenols in this environment, sodium acetate buffer (pH 3.3) was used as a solvent to explore the antioxidant capacity of six screened polyphenols (Section 3.2), compared with methanol. Table 4 shows the antioxidant capacities of six kinds of sugarcane vinegar polyphenols (CGA, CA, FA, L, SA, and PA) in methanol and sodium acetate solutions. The IC50 values of FA and SA in sodium acetate buffer (pH 3.3) were higher than those in methanol, indicating that methanol provides a more favorable environment for their antioxidant activities. Conversely, the IC50 values of PA, CA, L, and CGA in sodium acetate buffer (pH 3.3) were significantly lower than those in methanol, suggesting that these four compounds exhibit stronger antioxidant activities in sodium acetate buffer (pH 3.3). This could be explained by the enhanced stability of CGA, CA, and PA in acidic conditions [34]. Similarly, Jiang et al. [35] found that the antioxidant activity of cinnamic acid derivatives increased with decreasing pH, with a more pronounced increment for compounds containing -COOH groups. FA and SA, which also contain -COOH, exhibited lower antioxidant activity in both methanol and sodium acetate buffer (pH 3.3). This could be attributed to the influence of the number of -OH groups on the aromatic ring [36]. Hydroxycinnamic acids (e.g., ferulic acid, FA) and hydroxybenzoic acids (e.g., SA) with one -OH group have been found to exhibit weak antioxidant or pro-oxidant properties. On the other hand, the derivatives containing two or more -OH groups (CA and PA) have demonstrated enhanced antioxidant activity [37].

#### 3.3.2. Interactions Between the Antioxidants in Sugarcane Vinegar

Extracts are complex mixtures of phenolic compounds that participate in redox or free radical scavenging reactions, where these compounds may interact synergistically, antagonistically, or additively [38]. To further explore the interactions between antioxidant polyphenolic compounds in sugarcane vinegar aqueous extract, we assessed the DPPH scavenging capacity of binary combinations of these compounds to determine the Combination Index (CI_wt_). The results indicated that out of the 15 tested combinations of sugarcane vinegar polyphenolic compounds, all combinations, except L + PA, showed a CI_wt_ lower than methanol in sodium acetate buffer (pH 3.3) (Table 5). In the study, combinations like CGA + FA, CGA + L, CGA + PA, CGA + SA, FA + L, and L + PA displayed varying levels of antagonism in methanol. Still, they exhibited synergistic effects in sodium acetate buffer (pH 3.3). This could be attributed to the higher polarity of the pH 3.3 sodium acetate buffer compared to methanol [39]. In addition, it has been demonstrated that polyphenols could neutralize DPPH radicals in polar solvents, like methanol and water, through the SPLET (sequential proton loss–electron transfer) mechanism. In this process, the molecule’s ability to donate electrons increases with higher solvent polarity, leading to greater DPPH scavenging activity [33,40]. Therefore, sodium acetate buffer (pH 3.3) provided a more favorable environment than methanol for the antioxidant activity of sugarcane vinegar polyphenols and their mixtures.

CA + CGA, CA + L, CA + PA, and CA + SA exhibited antagonistic effects in both solvents, while CA + FA showed antagonism in methanol but additivity in sodium acetate buffer (pH 3.3). This suggests that their antioxidant activities are influenced by both the solvent and the compound structure. Of the six compounds tested, only CA contained the -OH group at position 3, suggesting that the -OH group at this position may promote the antagonistic effects of CA in combination with other polyphenolic compounds. Çelik and Gökmen [41] found that the position of -OH groups influenced the antioxidant activity of binary mixtures of hydroxycinnamic and hydroxybenzoic acids in liposomes. Specifically, if the -OH group is at position 4 of the compound, it exerts an antagonistic effect when the accompanying -OH group is in the interstitial position and a synergistic effect when it is in the neighboring position.

All the tested combinations demonstrated varying synergistic effects in sodium acetate buffer (pH 3.3) except those containing CA. In a previous study, our group found that sugarcane original vinegar could ease oxidative stress in mice [1]. However, the specific components and mechanism of action by which sugarcane vinegar exerts anti-oxidative stress remained unclear. Based on the results of this study, we hypothesize that the antioxidant stress effects of sugarcane vinegar may be attributed to the synergistic antioxidant effects of CGA, CA, FA, L, SA, and PA.

### 3.4. Network Pharmacology

#### 3.4.1. Potential Targets of Active Antioxidants and Compound Network Analysis

**Figure 3:**
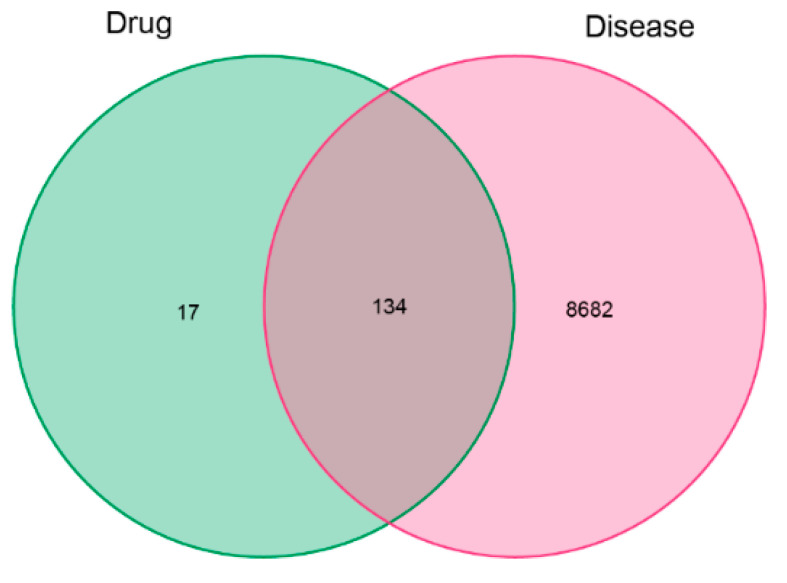
Venn diagram of sugarcane vinegar polyphenols and oxidative stress targets (“Drug” represents sugarcane vinegar polyphenols, and “Disease” represents targets).

The targets of six sugarcane vinegar polyphenols (CGA, CA, FA, L, PA, and SA) were predicted using the Swiss Target Prediction and TCMSP databases. The results from both databases were combined to remove duplicates, resulting in 151 potential targets. Oxidative-stress-related targets were then identified using the GeneCards, OMIM, PharmGKB, TTD, and DrugBank databases, yielding 8816 targets after screening. The Venn diagram analysis of sugarcane vinegar active polyphenol targets and oxidative-stress-related targets revealed a total of 134 intersecting targets (Figure 3), which represent the potential targets through which sugarcane vinegar polyphenols may exert their anti-oxidative stress effects.

**Figure 4:**
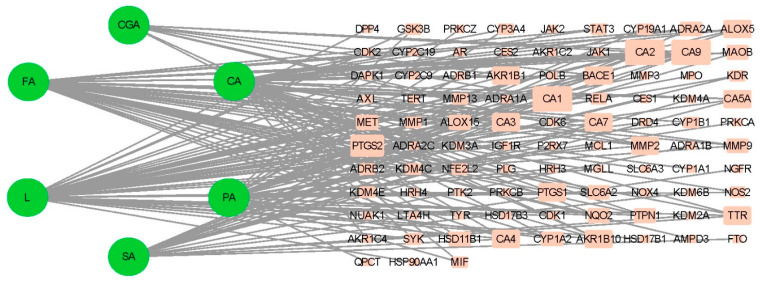
Network diagram of sugarcane vinegar active antioxidants and their target genes to combat oxidative stress (circles represent antioxidants, and rectangles represent target genes: larger the rectangle, higher the node degrees).

The compound–target network diagram of sugarcane vinegar polyphenol compounds–oxidative stress targets (Figure 4) was constructed using Cytoscape 3.7.0 software and analyzed topologically. In this diagram, the more edges a node has, the more targets it interacts with, making it more likely to be a core component. The six sugarcane vinegar polyphenols were ranked in descending order of the number of edges as follows: L > PA > FA > CA > SA > CGA. The rectangles represent the targets, with larger rectangles indicating higher node degrees and a greater likelihood of being core targets, while smaller ellipses indicate the opposite. The figure shows that the nodes with larger degree values are mainly Carbonic Anhydrase 9 (CA9), Carbonic Anhydrase 1 (CA1), Carbonic Anhydrase 2 (CA2), Prostaglandin-Endoperoxide Synthase 2 (PTGS2), and Prostaglandin-Endoperoxide Synthase 1 (PTGS1). These targets play a crucial role in a series of interconnected reactions closely associated with oxidative stress. For instance, Gryko et al. [27] reported that coumarin derivatives can reduce inflammation induced by oxidative stress by inhibiting CA9. Additionally, studies have identified PTGS2 as a significant oxidative-stress-related biomarker linked to the failure of arteriovenous fistulas [42]. Moreover, the compound network diagram indicated that the antioxidants in sugarcane vinegar have a synergistic, multi-component, and multi-targeted anti-oxidative potential.

#### 3.4.2. Characteristic Analysis of Antioxidant Target Protein Pathway Network

**Figure 5:**
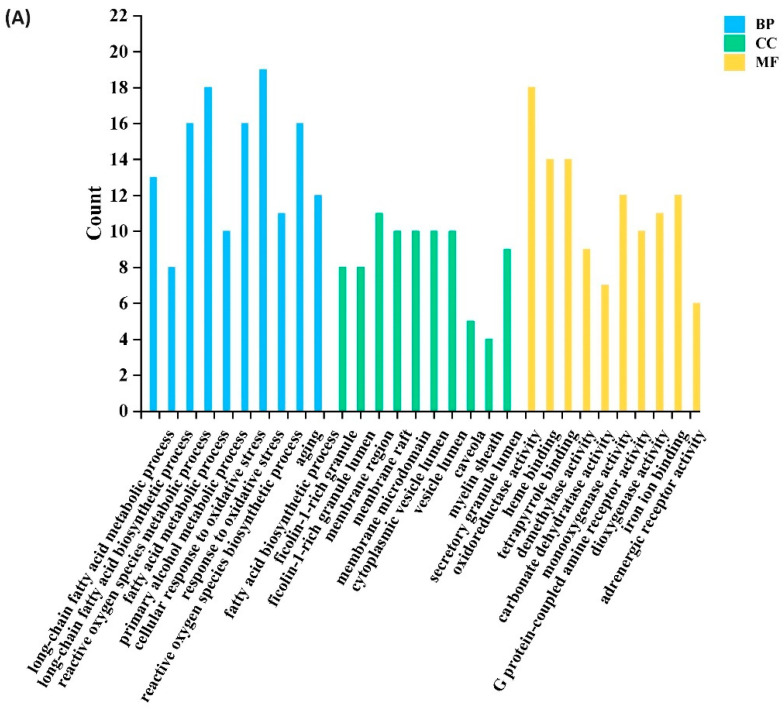

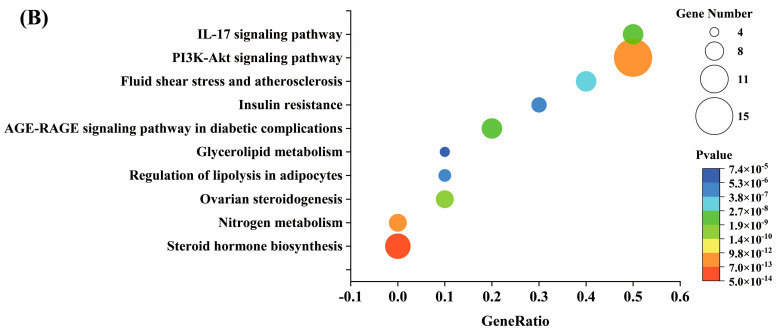
The GO and KEGG enrichment analysis of sugarcane vinegar polyphenols to combat oxidative stress. (**A**) The bar graph of the top 10 GO terms, including biological process (BP), cellular compound (CC), and molecular function (MF). (**B**) The bubble diagram of the top 10 KEGG pathways.

A GO functional enrichment analysis was conducted on the 134 intersection targets of sugarcane vinegar polyphenols and oxidative stress. The analysis yielded 732 entries related to biological processes (BP), 25 entries related to cellular components (CC), and 120 entries related to molecular functions (MF). The top 10 entries closely associated with oxidative stress in each category (BP, CC, and MF) were selected for visualization (Figure 5A) based on their corrected *p*-values. For example, the biological processes (BPs) primarily included the fatty acid biosynthetic process, reactive oxygen species biosynthetic process, response to oxidative stress, cellular response to oxidative stress, and reactive oxygen species metabolic process. Regarding cellular components (CCs), significant entries included ficolin-1-rich granule, myelin sheath, ficolin-1-rich granule lumen, membrane region, and membrane microdomain. In terms of molecular functions (MFs), the enriched terms mainly involved heme binding, adrenergic receptor activity, iron ion binding, dioxygenase activity, monooxygenase activity, and oxidoreductase activity. For instance, Triptriolide (T11) has been shown to exert antioxidant effects by modulating the activities of oxidoreductase and heme oxygenase in Triptolide (T9)-induced human kidney 2 (HK2) cells and BALB/c mice [43]. These findings effectively demonstrate that sugarcane vinegar polyphenols alleviate oxidative stress damage by modulating biological processes across multiple cellular compartments. KEGG pathway enrichment analysis revealed that the intersection targets of sugarcane vinegar polyphenols and oxidative stress were significantly enriched in 173 pathways (*p* < 0.05). The top 10 pathways associated with oxidative stress were then selected for visualization (Figure 5B) These pathways primarily involve the PI3K-Akt signaling pathway, steroid hormone biosynthesis, IL-17 signaling pathway, fluid shear stress and atherosclerosis, and other related pathways. Notably, the PI3K-Akt signaling pathway, fluid shear stress and atherosclerosis, regulation of lipolysis in adipocytes, and triglyceride metabolism are closely related to hyperlipidemia [44,45,46,47]. The PI3K/Akt axis is a crucial signaling pathway regulating cellular proliferation, differentiation, senescence, and apoptosis across various cell types [48,49]. For example, recent research suggests that L alleviates H_2_O_2_-induced oxidative stress by increasing the expression of p-PI3K and p-AKT. Studies have demonstrated that quince extract significantly increases the expression levels of p-PI3K and p-AKT in atherosclerotic mice, and its hypolipidemic effect was related to the PI3K/AKT signaling pathway [44]. This aligns well with our previous findings on the significant anti-oxidative stress and hypolipidemic effects of sugarcane vinegar on hyperlipidemic mice [1]. That leads us to propose that the anti-oxidative stress and hypolipidemic effects of sugarcane vinegar may stem from its polyphenols’ modulation of these pathways.

**Figure 6:**
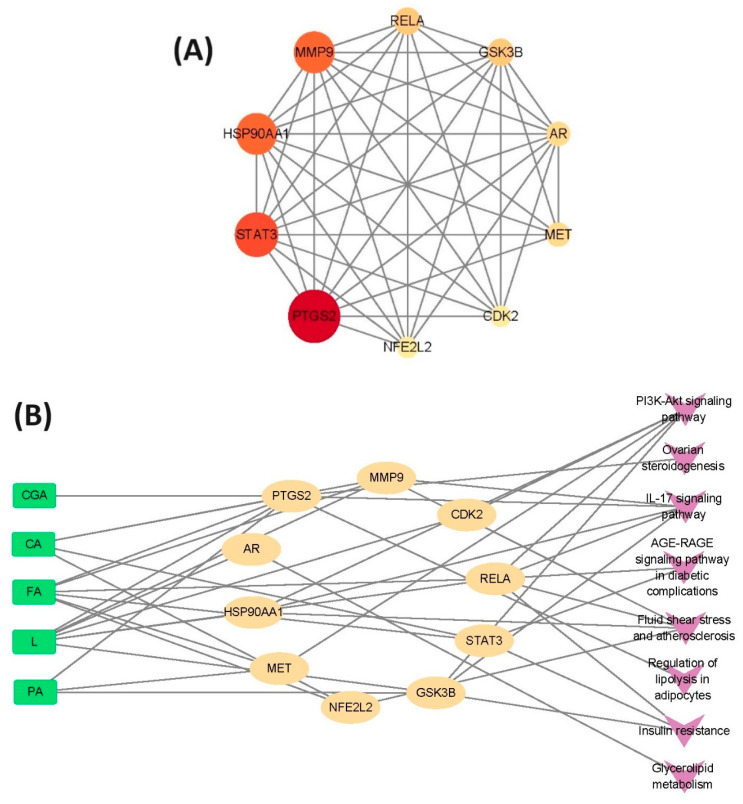
The PPI network analysis of core targets screened from potential targets (**A**) and network diagram of the active component–key target pathway (**B**) of sugarcane vinegar polyphenols to mitigate oxidative stress. Note: The full names of the targets are as follows: Androgen Receptor (AR), Cyclin-Dependent Kinase 2 (CDK2), Glycogen Synthase Kinase 3 Beta (GSK3B), Heat Shock Protein 90 Alpha Family Class A Member 1 (HSP90AA1), MET Proto-Oncogene, Receptor Tyrosine Kinase (MET), Matrix Metallopeptidase 9 (MMP9), NFE2-Like BZIP Transcription Factor 2 (NFE2L2), Prostaglandin-Endoperoxide Synthase 2 (PTGS2), RELA Proto-Oncogene, NF-κB Subunit (RELA), and Signal Transducer and Activator of Transcription 3 (STAT3).

A total of 10 core targets of sugarcane vinegar polyphenols acting on oxidative stress were identified based on topological parameters (Figure 6A): Prostaglandin-Endoperoxide Synthase 2 (PTGS2), Signal Transducer and Activator of Transcription 3 (STAT3), Heat Shock Protein 90 Alpha Family Class A Member 1 (HSP90AA1), Matrix Metallopeptidase 9 (MMP9), NF-KB Subunit (RELA), Glycogen Synthase Kinase 3 Beta (GSK3B), Androgen Receptor (AR), MET Proto-Oncogene, Receptor Tyrosine Kinase (MET), Cyclin-Dependent Kinase 2 (CDK2), and NFE2-Like BZIP Transcription Factor 2 (NFE2L2). In the network diagram, a larger and darker-colored circle represents a node with a higher degree value, indicating that the node occupies a core position. The results showed that the targets of sugarcane vinegar polyphenols related to oxidative stress were distributed across multiple pathways (Figure 6B). This reflects the multi-component, multi-target, and multi-pathway nature of the antioxidant mechanism of sugarcane vinegar. In descending order of core target enrichment, the signaling pathways appeared as PI3K-Akt signaling pathway = IL-17 signaling pathway > fluid shear stress and atherosclerosis > insulin resistance > ovarian steroidogenesis = regulation of adipocyte lipolysis = glycerol ester metabolism. This analysis revealed 10 core targets (PTGS2, STAT3, HSP90AA1, MMP9, RELA, GSK3B, AR, MET, CDK2, and NFE2L2) through the PPI network. However, no targets were identified for syringic acid (SA) so it was not included in subsequent analyses. Among the five sugarcane vinegar polyphenol compounds (CGA, CA, FA, L, PA), L affected the most signaling pathways, followed by FA. Both L and FA acted on seven signaling pathways, which is consistent with their strong synergistic antioxidant effect in sodium acetate buffer (pH 3.3), as shown in Table 4. The documented literature indicates that luteolin protects PC-12 cells from H_2_O_2_-induced oxidative injury by activating the PI3K/AKT pathway [50]. Furthermore, phosphatidylinositol 3-kinase (PI3K) is a central regulator of aging, and ferulic acid has been shown to alleviate oxidative stress and delay the aging process by inhibiting PI3K [51,52]. This affirms that luteolin and ferulic acid in sugarcane vinegar synergistically scavenge free radicals and act on the same signaling pathways to circumvent oxidative stress.

**Figure 7:**
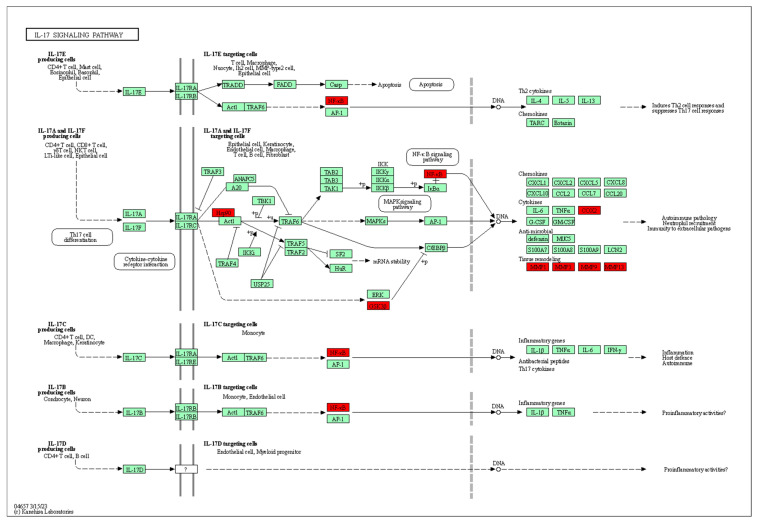
The map of the IL-17 signaling pathway illustrating the enrichment of 11 target genes (red color).

The interleukin 17 (IL-17) family, a subset of cytokines consisting of IL-17A-F, plays a key role in acute and chronic inflammatory responses [53]. During an inflammatory response, the expression of downstream inflammatory factors such as IL-6, TNF-α, COX2, and MMP9 increases, and downregulating these factors inhibits the activation of the IL-17 signaling pathway [54]. As shown in Figure 7, L and FA can directly act on MMP9 or affect its downstream inflammatory factors by acting on RELA (NF-κB p65) as the target. There is a mutually reinforcing relationship between oxidative stress and the inflammatory response; thus, modulating associated inflammatory factors can reduce levels of ROS and MMP1 [55]. MDA, a lipid peroxide produced by unsaturated fatty acids in cell membranes when attacked by ROS, is an important indicator of oxidative stress. Ref. [1] found that supplementing hyperlipidemic mice with sugarcane vinegar significantly reduced MDA levels and alleviated oxidative stress symptoms. Combined with the results of the present study, it can be concluded that the modulation of the IL-17 signaling pathway by phenolic compounds such as L and FA may be one of the mechanisms behind sugarcane vinegar’s anti-oxidative stress activity.

### 3.5. Molecular Docking of Active Antioxidants and Key Targets

**Figure 8:**
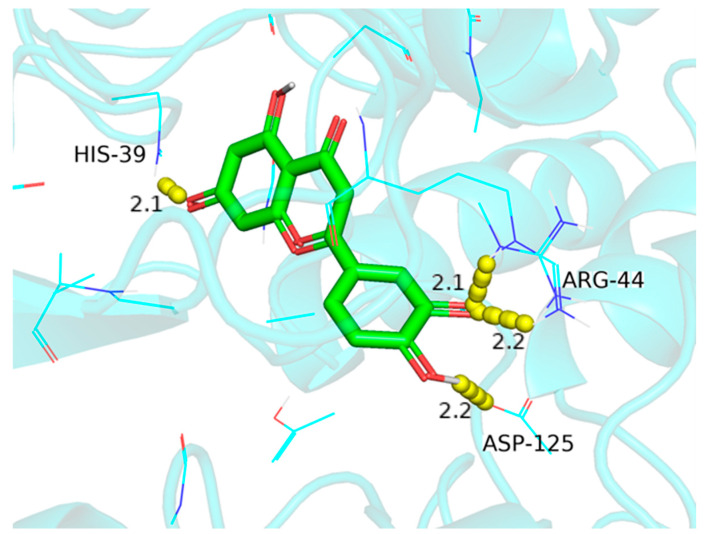
The molecular docking pattern of luteolin (L) with PTGS2.

The molecular docking method can be applied to effectively analyze molecular interactions. In this study, 5 sugarcane vinegar polyphenols (CGA, CA, FA, L, PA) and 10 core targets (PTGS2, STAT3, HSP90AA1, MMP9, RELA, GSK3B, AR, MET, CDK2, and NFE2L2) underwent molecular docking, and the results are presented in Table 6. The minimum binding energies ranged from −10 kcal/mol to −5.1 kcal/mol, and the free energies of binding were less than −5.0 kcal/mol, indicating a good binding affinity between the compounds and targets [15]. The hydrogen bonding distances ranged from 0.8 to 2.9 Å, which is shorter than conventional hydrogen bonds (3.5 Å), indicating a stable binding conformation [56]. Among the compounds, L exhibited the least binding energy to PTGS2 at −10 kcal/mol. The potential binding sites were the amino acid residues ARG-44, ASP-125, and HIS-39, with hydrogen bonding distances of 2.2 Å, 2.2 Å, and 2.1 Å, respectively. This suggests that the binding conformation between them may be the most stable, and the binding pattern is plotted in Figure 8. Both L and FA can bind at the same positions on the same target, such as ARG-97, ARG-162, and ALA-164 of MMP9. Additionally, L and FA can bind at the same positions on ARG-208, ARG-358, and ASP-309 of RELA (NF-κB), and ARG-44 of PTGS2. Previous studies have shown that luteolin and ferulic acid can act on MMP9, NF-κB, PTGS2, and other targets to regulate the IL-17 signaling pathway, thereby reducing inflammation [57,58]. This further suggests that sugarcane vinegar may exert its anti-oxidative stress effect on the IL-17 signaling pathway through the combined action of L and FA, consistent with the findings of Section 3.3.2 and Section 3.4.2. Nonetheless, relevant studies investigating the synergistic antioxidant and anti-inflammatory effects of luteolin and ferulic acid through the regulation of the IL-17 signaling pathway were not found, highlighting the need for further research and validation in this area.

## 4. Conclusions

This study delved into the anti-oxidative value of sugarcane vinegar, providing deep insights into the antioxidant capabilities of its constituent phenolics in both single and binary systems, along with the corresponding molecular mechanisms in combating oxidative stress. The results showed a strong positive correlation between TPC, TFC, and the DPPH and ABTS scavenging capacities of sugarcane vinegar extracts using different solvent systems. The six main active components (CA, FA, L, PA, SA, and CGA) of sugarcane vinegar polyphenols exhibiting antioxidant activity were screened using DPPH-UPLC-MS. Strong antioxidant capacities (PA, CA, L, and CGA) were observed in a sugarcane simulated environment as compared to organic systems, which explains the hydrophilic nature of antioxidants. In addition, the binary combinations of the six sugarcane vinegar polyphenols demonstrated stronger synergistic antioxidant effects in sodium acetate buffer (pH 3.3) than in methanol, with the L + FA combination exhibiting the most significant synergistic effect. The core targets of sugarcane vinegar polyphenols in mitigating oxidative stress were primarily PTGS2, STAT3, HSP90AA1, MMP9, RELA, GSK3B, AR, MET, CDK2, and NFE2L2. These targets are directly involved in the frontline response to oxidative stress, fatty acid metabolism process, cellular response to oxidative stress, ROS metabolism process, and senescence. The free energies of binding these targets to the ligands (CGA, CA, FA, L, PA) are in the range of −10 kcal/mol to −5.1 kcal/mol. In addition, hydrogen bonding is present in all binding conformations, suggesting they can bind together well. Molecular evidence suggests that sugarcane vinegar exerts its antioxidant effects through key components such as CGA, CA, FA, L, and PA, which bind to specific molecular targets. These compounds influence several critical signaling pathways, including the PI3K-Akt pathway, IL-17 signaling pathway, and pathways associated with fluid shear stress and atherosclerosis, contributing to its potential antioxidant activity. Thus, sugarcane vinegar can be considered a valuable byproduct of sugarcane with potential applications as a functional food capable of mitigating oxidative stress in biological systems. In the future, the findings from these antioxidant interactions can be utilized in developing and formulating functional sugarcane vinegar products. The underlying mechanisms of action should be further confirmed through cellular and animal studies, along with other advanced experimental approaches.

## Figures and Tables

**Table 1:** Description and symbols of synergism or antagonism in sugarcane vinegar polyphenol combination studies analyzed with the combination index method [21].

Range of Combination Index (CI_wt_)	Description	Assigned Symbols
0.1–0.3	Strong synergism	+ + + +
0.3–0.7	Synergism	+ + +
0.7–0.85	Moderate synergism	+ +
0.85–0.9	Slight synergism	+
0.9–1.10	Nearly additive	±
1.10–1.20	Slight antagonism	−
1.20–1.45	Moderate antagonism	− −
1.45–3.3	Antagonism	− − −
3.3–10	Strong antagonism	− − − −

**Table 2:** Correlation analysis of TEAC, TPC, and TFC of sugarcane vinegar extract.

Correlation Coefficient (R^2^)	TPC	TFC	TEAC	
			DPPH	ABTS
TPC	1			
TFC	0.983 *	1		
DPPH	0.966 *	0.968 *	1	
ABTS	0.970 *	0.978 *	0.991 *	1

Note: * statistical significance at *p*-value < 0.01.

**Table 3:** The polyphenol content in sugarcane vinegar extract before and after reacting with DPPH.

No.	Compound	T_R_ (min)	Before Reaction (mg/L)	After Reaction (mg/L)	Limit of Quantification (mg/L)	Consumption Rate (%)	Antioxidant Activity
1	AG	9.15	0.1	0.092	0.0004	8.00	×
2	CA	4.55	0.196	0.16	0.03	18.37	√
3	CMA	8.97	0.052	0.056	0.01	−7.69	×
4	pCA	5.99	7.414	7.682	0.03	−3.61	×
5	FA	6.74	12.497	2.121	0.08	83.03	√
6	L	8.61	0.088	0.006	0.004	93.18	√
7	PA	2.83	6.302	1.594	0.05	74.71	√
8	SA	4.75	9.467	2.555	0.07	73.01	√
9	CGA	3.70	0.106	-	0.05	>95.28	√
10	VA	4.57	2.404	2.338	0.08	2.75	×

Notes: “-”content is either below the limit of quantification or not detected, “×” no antioxidant activity or insignificant antioxidant activity, “√” potential antioxidant activity.

**Table 4:** Effect of solvent system on antioxidant activity of polyphenols from sugarcane vinegar.

No.	Compound	IC_50_ (µg/mL)	
		Methanol	Sodium Acetate Buffer (pH 3.3)
1	CGA	25.59	10.22
2	CA	11.18	5.59
3	FA	23.29	25.56
4	L	9.84	7.28
5	SA	15.17	16.6
6	PA	11.26	5.95

**Table 5:** CI_wt_ values of binary combinations of antioxidants in methanol and sodium acetate buffer accessed by the scavenging of DPPH.

No.	Combinations	Methanol		Sodium Acetate Buffer (pH 3.3)	
		CI_wt_	Assigned Symbol	CI_wt_	Assigned Symbol
1	CGA + CA	2.1	− − −	1.16	−
2	CGA + FA	1.73	− − −	0.43	+ + +
3	CGA + L	2.46	− − −	0.42	+ + +
4	CGA + PA	6.23	− − − −	0.55	+ + +
5	CGA + SA	2.99	− − −	0.49	+ + +
6	CA + FA	2.62	− − −	1.04	±
7	CA + L	2.36	− − −	1.49	− − −
8	CA + PA	2.18	− − −	1.69	− − −
9	CA + SA	1.85	− − −	2.79	− − −
10	FA + L	1.29	− −	0.19	+ + + +
11	FA +PA	0.74	+ +	0.31	+ + +
12	FA + SA	0.31	+ + +	0.23	+ + + +
13	L + PA	1.52	− − −	0.31	+ + +
14	L + SA	0.49	+ + +	0.34	+ + +
15	PA + SA	0.53	+ + +	0.45	+ + +

Notes: The assigned symbols follow the pattern developed by [21].

**Table 6:** Molecular docking simulation for active molecules of sugarcane vinegar extract and their targets.

No.	Molecule Name	Target	Residues Involved in H Bonding	H-Bond Length (Å)	Docking Energy (kcal/mol)
1	CGA	PTGS2	TYR373, PHE-371, SER-126, GLN-372	2.2, 2.1, 2.1, 2.9	−8.9
2	CA	NFE2L2	ILE-33, VAL-37	2.5, 2.5	−5.7
3	CA	STAT3	LYS-370	1.9	−5.9
4	CA	MMP9	HIS-91, GLN-126, TYR-92, TYR-98, VAL-172	2.2, 2.6, 2.0, 2.4, 2.0	−6.9
5	CA	PTGS2	GLN-374, TYR-373, ASN-537, VAL-228	2.5, 2.5, 2.2, 2.6	−6.9
6	FA	MET	MET-1160	2.4	−6.9
7	FA	NFE2L2	PHE-35	2.8	−5.5
8	FA	STAT3	ASP-570, GLN-511	2.3, 2.7	−5.8
9	FA	RELA	ARG-208, ARG-358, ASP-309	2.4, 1.9, 2.4	−5.8
10	FA	MMP9	ARG-97, ARG-162, ALA-164	2.5, 2.7, 2.8	−6
11	FA	PTGS2	ARG-44, ARG-469, GLY-45, GYS-47	2.4, 2.2, 2.2, 2.1	−6.6
12	L	AR	GLN-711, ARG-752	2.5, 2.1	−9.6
13	L	HSP90AA1	PHE-138, GLY-135, GLY-137	2.3, 2.4, 2.8	−8.1
14	L	GSK3B	ARG-209, VAL-208, GLU-211, ASN-213	2.4, 2.7, 2.9, 0.8	−8.3
15	L	RELA	ARG-208, GLU-359, ARG-358, ASP-309	2.7, 2.0, 2.6, 2.1	−7.7
16	L	MMP9	ARG-97, ARG-162, ASP-103, ALA-164, GLY-195	2.7, 2.8, 2.3, 2.1, 2.7	−8.5
17	L	CDK2	HIS-84, ASP-86, LYS-89	2.5, 2.4, 2.4	−8.9
18	L	PTGS2	ARG-44, ASP-125, HIS-39	2.2, 2.2, 2.1	−10
19	PA	MET	ASP-1222	2.6	−5.5
20	PA	GSK3B	THR-289	2.3	−5.1
21	PA	PTGS2	GLY-45	1.9	−5.6

## Data Availability

The original contributions presented in the study are included in the article, further inquiries can be directed to the corresponding author.
